# A Remarkable Case of Micro-Endemism in *Laonastes aenigmamus* (Diatomyidae, Rodentia) Revealed by Nuclear and Mitochondrial DNA Sequence Data

**DOI:** 10.1371/journal.pone.0048145

**Published:** 2012-11-14

**Authors:** Violaine Nicolas, Vincent Herbreteau, Arnaud Couloux, Kham Keovichit, Bounneuang Douangboupha, Jean-Pierre Hugot

**Affiliations:** 1 UMR CNRS 7205, Département Systématique et Evolution, Muséum National d'Histoire Naturelle, Paris, France; 2 IRD, UMR ESPACE-DEV, Montpellier, France; 3 Genoscope, Centre National de Séquençage, Evry, France; 4 National Agriculture And Forestry Research Institute (NAFRI), Vientiane, Lao PDR; Texas A&M University, United States of America

## Abstract

*L. aenigmamus* is endemic to the limestone formations of the Khammuan Province (Lao PDR), and is strongly specialized ecologically. From the survey of 137 individuals collected from 38 localities, we studied the phylogeography of this species using one mitochondrial (Cyt b) and two nuclear genes (BFIBR and GHR). Cyt b analyses reveal a strong mtDNA phylogeographical structure: 8 major geographical clades differing by 5–14% sequence divergence were identified, most of them corresponding to distinct karst areas. Nuclear markers display congruent results but with a less genetic structuring. Together, the data strongly suggest an inland insular model for *Laonastes* population structure. With 8 to 16 evolutionary significant units in a small area (about 200×50 km) this represents an exceptional example of micro-endemism. Our results suggest that *L. aenigmamus* may represent a complex of species and/or sub-species. The common ancestor of all *Laonastes* may have been widely distributed within the limestone formations of the Khammuan Province at the end of Miocene/beginning of the Pliocene. Parallel events of karst fragmentation and population isolation would have occurred during the Pleistocene or/and the end of the Pliocene. The limited gene flow detected between populations from different karst blocks restrains the likelihood of survival of *Laonastes*. This work increases the necessity for a strict protection of this rare animal and its habitat and provides exclusive information, essential to the organization of its protection.

## Introduction

Eroded limestone outcrops form a prominent part of the landscape in Southeast Asia. Because of high limestone weathering [1], [2] erosion in these areas resulted in scattered, isolated limestone hills with steep flanks called karsts towers. Despite a high diversity of habitat specialists and endemic taxa [3]–[9], these limestone karsts remain among the least studied ecosystems in Southeast Asia. Between 1985 and 2004 they contributed only to 1% of the global and regional biodiversity research output from terrestrial and freshwater ecosystems, while they cover around 10% of the land area in Southeast Asia [4]. However, in the Khammuan Province of Lao PDR, several new endemic vertebrate species were recently described: a bird, *Pycnonotus hualon* [3], a bat, *Hipposideros scutinares* [10], a gymnure, *Hylomys megalotis* [11], and a murid rodent, *Saxatilomys paulinae* [12].

*Laonastes aenigmamus*, recently described from this region by Jenkins *et al.* [13], is of special interest. Using comparative morphological and molecular data, this new species was initially described as a member of a new genus and ranked within the Hystricognathi rodents in a new family: the Laonastidae [13]. But, further studies demonstrated: first, that *L. aenigmamus* is a member of the Diatomyidae, a fossil family known from early Oligocene to late Miocene in Pakistan, India, Thailand, China, and Japan [14]; second, that the Diatomyidae are the sister group of the Ctenodactylidae, a family of small rodents found in rocky deserts across the northern parts of Africa; third, that together with the Hystricognathi, the Diatomyidae and the Ctenodactylidae form the suborder Ctenohystrica [15].

In a previous study we presented a preliminary analysis of the genetic diversity of *L. aenigmamus* [16]: 52 specimens were sampled and the population structure was surveyed by sequencing 887 base pairs of the Cytochrome *b* (Cyt *b*) gene. Phylogenetic and haplotypic network reconstructions revealed three well-supported and rather divergent lineages, suggesting that *L. aenigmamus* populations are geographically structured. However, altogether these samples represented a limited part of the estimated range of the species; most of them were sampled in local markets and their exact geographic origin was impossible to determine; this study was based on a fragment of a single mitochondrial gene.

Before its scientific discovery, the kanyou was considered as a kind of game, trapped and eaten by the villagers. Since 2008, the conservation of *L. aegnimamus* is regulated in Lao PDR and since 2009 this species is listed as «Endangered» on the IUCN Red List. During the last four years we add the unique opportunity to accompany local officers in the field when they explained this change to local populations, and to sample for molecular analyses the last specimens that were captured by traditional hunters. We were able to sample 137 individuals for which the exact locality of collect was known. It represents 38 localities probably spanning the whole geographical range of the species. Thus, the sampling obtained for this study is exceptional both in terms of number of individuals and number of localities sampled and will not be able to be carried out again. In the present study we: (1) improve evaluation of the genetic diversity and delineation of the phylogeographical structure of *L. aenigmamus*, by sequencing both nuclear and mitochondrial markers of a larger number of geographically referenced individuals; (2) obtain a better definition of the geographical distribution of the species; (3) highlight the spatial and temporal history of the limestone formation of the Khammuan Province, using the evolutionary history of this species as a guide. Our analysis strongly underlines the necessity for a strict protection of this rare animal and its habitat and provides exclusive information, essential to the organization of its protection.

## Materials and Methods

### Ethics Statement

Since November 12th, 2008, the conservation of *L. aegnimamus* is regulated in Lao PDR (Circular No. 0963/PAFO, from the Khammuan Province, Agriculture and Forestry Office). Since January 12th, 2009, this species is listed as «Endangered» on the IUCN Red List. To realize this study, we obtained an exceptional letter of authorization (N° 1183 June 09th, 2008) from the Lao Government (Ministry of Planning and Investment). In December 2008, March 2009, November 2009, 2010 and 2011, five field sampling trips took place in the Khammuan Province in collaboration with the Lao National Agriculture and Forestry Research Institute (NAFRI). The Khammuan Province Agriculture and Forestry Office (PAFO) validated our field collection schedule and an officer escorted us. The protocol was approved by the French National Museum of Natural History (PPF 2011 “Biodiversité actuelle et fossile. Crises, stress, restaurations et panchronisme: le message systématique”).

All the specimens used in this study were captured by traditional hunters. When an animal was captured alive all efforts were made to minimize suffering (animals were maintained quietly in appropriate caging with enough food and moisture and adequate temperature), and it was handled under the guidelines of the American Society of Mammalogists [17]. Alive animals were euthanized by the injection of a lethal dose of isofluorane, followed by cervical dislocation.

### Specimens examined and mitochondrial DNA sequencing

**Figure 1:**
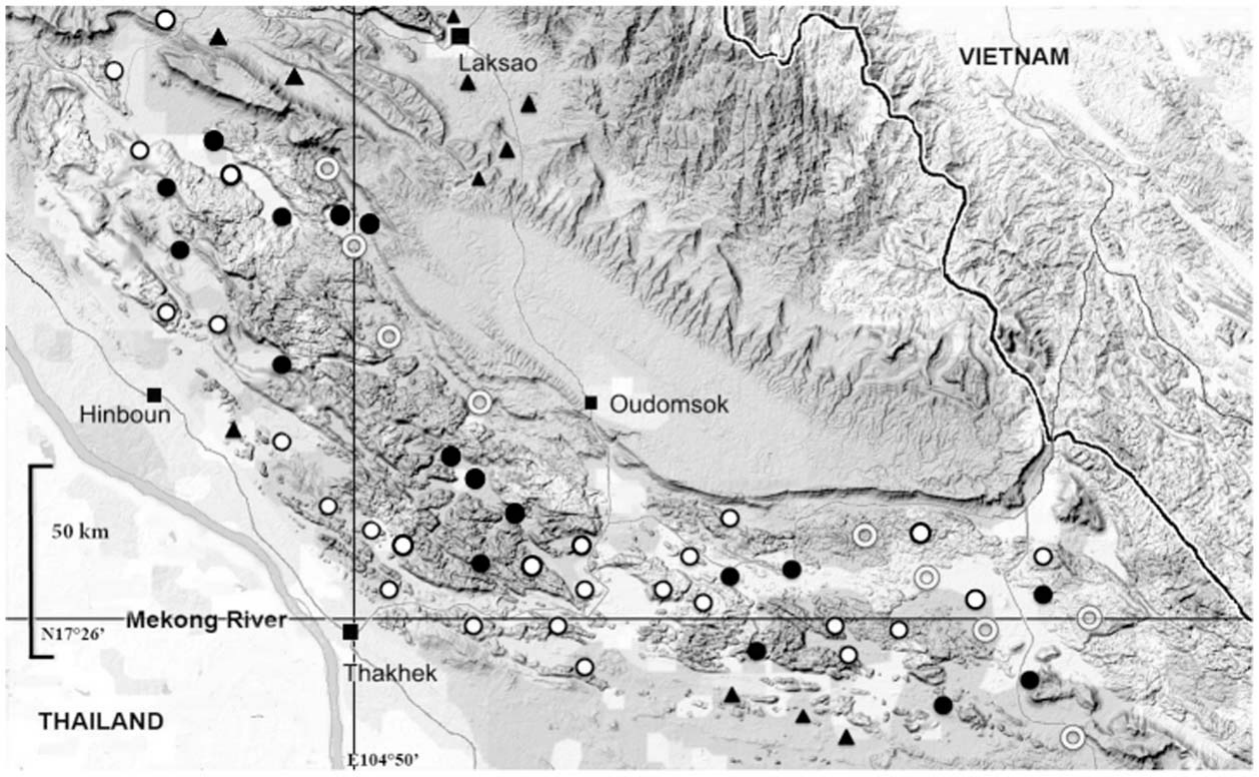
Geographical distribution of *L. aenigmamus* (plotted on a GoogleMap background). Circles indicate localities where the species certainly exists; white circles: localities for which samples are included in the present study; black circles: localities visited but where no sample could be obtained. Triangles: localities where *L. aenigmamus* is absent according to villager's. Rings: localities not investigated during recent field collections, but where the species is very probably present.

Specimens whom geographical provenance was not precisely established were excluded. The present study includes 137 individuals representing 38 localities (Fig. 1; Table 1; see Table S1 for the list of specimens used in this study, Genbank numbers and Museum numbers). DNA was extracted from ethanol-preserved tissues by the CTAB method [18]. The Cyt *b* gene was amplified for 135 specimens using polymerase chain reaction (PCR) primers L14723 and H15915 [19]. PCR conditions were the same as those described in Nicolas et al. [20]. We also amplified and sequenced two nuclear gene fragments: the Intron 7 of the beta-fibrinogen (BFIBR) and the exon 10 of the growth hormone receptor (GHR). Amplification and sequencing of these two nuclear genes were performed for 83 specimens and 80 specimens for BFIBR and GHR genes respectively (for technical reasons amplification of some individuals for one gene or the other failed), representing all known mtDNA groups. For the BFIBR we used the primers BFIBR1 and BFIBR2 published by Seddon et al. [21], and an annealing temperature of 50°C. For the GHR we used the primers GHR1 and GHR2 published by Adkins et al. [22] and the primers GHR7 and GHR8 published by Lecompte et al. [23], and an annealing temperature of 55–60°C. The double-stranded PCR products were purified and sequenced at the Genoscope (Ivry/Seine, France). Sequences were aligned by eye. Sequences were sublitted to the Genbank database (HQ687370 to HQ687474, and JN796953 to JN797145).

**Table 1:** Details of the number of specimens sequenced per gene and per collection site.

				Nb specimens sequenced			
Collection site	Latitude	Longitude	mt DNA Clades	Cytb	BFIBR	GHR	Total
Ban Dang, site 1	17.480°	105.262°	D1,D2	8	4	4	8
Ban Dang, site 2	17.465°	105.251°	D2	1	1	1	1
Ban Doye	17.558°	104.844°	B2	2	2	2	2
Ban Koun Gneun, site 1	18.171°	104.450°	F	2	2	2	2
Ban Koun Gneun, site 2	18.183°	104.460°	F	2	2	1	2
Ban Koun Gneun, site 3	18.165°	104.482°	F	1	1	1	1
Ban Mann, site 1	17.393°	105.473°	D2	4	1	1	4
Ban Mann, site 2	17.405°	105.539°	D2	2	2	2	2
Ban Mann, site 3	17.441°	105.445°	D2	1	1	1	2
Ban Muang	17.554°	104.834°	B1,B2	3	2	2	3
Ban Muangueua	18.051°	104.748°	E1,E3	3	3	3	3
Ban Na Muang	17.821°	104.644°	E2	3	3	3	3
Ban Nam Dik1	17.601°	104.747°	B1	1	1	1	1
Ban Nam Dik2	17.638°	104.733°	A5	4	3	2	4
Ban Nasae	17.371°	105.087°	A4	3	3	3	3
Ban Natangchaye	17.457°	105.608°	D2	2	2	2	2
Ban NaTung	17.476°	105.107°	C	1	1	1	1
Ban Nonetoum	17.531°	105.259°		0	1	1	1
Ban Phakonko	18.086°	104.511°	E1	1	1	1	1
Ban Phalem	17.575°	104.813°	B1	4	4	4	4
Ban Phon Saet, site 1	17.511°	105.244°	D1,D2	4	4	4	4
Ban Phon Saet, site 2	17.527°	105.271°	D1	2	1	1	2
Ban Thalak	17.554°	105.628°	H	1	1	0	1
Ban Thapa, site 1	17.537°	105.247°	D1,D2	3	2	2	3
Ban Thapa, site 2	17.555°	105.263°	D1,D2	13	5	5	13
Ban Thok	17.472°	104.917°	A3	3	3	3	3
Ban Vong Khon	17.513°	105.718°	G	2	2	2	2
Between Ban Thong Kong and Ban Phonkeo	17.516°	105.387°	D2	2	2	2	2
Khok Sawang, site 1	17.481°	105.143°	C	1	1	1	1
Khok Sawang, site 2	17.508°	105.193°	D1,D2	3	3	3	3
Nahin	18.203°	104.495°	F	2	2	2	2
Phon Savanh North	17.483°	105.084°	C	1	1	1	1
Phon Savanh-Km1	17.440°	105.084°	A1	6	3	3	6
Phon Savanh-Km3	17.435°	105.099°	A1	15	5	5	15
Phon Savanh-Km5	17.431°	105.107°	A1	26	5	5	26
Pi Peng	17.828°	104.575°	E1	1	1	1	1
Thamel	17.500°	104.874°	A2	2	2	2	2
Total				135	83	80	137

See Supplementary material for GenBank numbers and specimen numbers.

The “numts” are usually characterized by the presence of indels, stop codons, frame-shift mutations and amplification of heterozygotes [24], [25]. We have not observed any of these indications in our dataset. Moreover the base composition bias per codon position was not significantly different between individuals. The strongest compositional bias for all individuals was observed at the 3^rd^ codon position (A = 36%, C = 46%, G = 4%, T = 14%), and the least at the first codon position (A = 27%, C = 26%, G = 22%, T = 25%). This is congruent with the results obtained for other vertebrates species [26]. So we believe that numts were not present in our dataset

### Phylogenetic analyses

Evolutionary relationships among sequences were estimated by constructing phylogenetic trees using maximum parsimony (MP), maximum likelihood (ML) and Bayesian Markov chain Monte Carlo phylogenetic analyses (MCMC). MP analysis was performed with PAUP 4b10 [27], ML analysis with PHYML [28], and Bayesian analysis with MrBayes 3.1.2 [29].

The MP analysis was performed with tree-bisection-reconnection (TBR) branch swapping option and 10 random addition replicates. We estimated the robustness of internal nodes by 1,000 bootstrapping replicates (each with a single replication of random addition of taxa). An equal weighting of character-state transformations was applied.

MrModeltest 3.04 [30] was used to evaluate the fit of 24 nested models of nucleotide substitution to the data. According to Akaike information criterion, MrModeltest recommended the GTR+I+G model for the Cytb dataset, GTR+G for the BFIBR dataset and HKY+G for the GHR dataset. These models were used in ML and Bayesian analyses. ML analyses were performed on the PHYML online web server [31] using the BIONJ distance-based tree as starting tree. Bootstrap analysis (500 replicates) was used to estimate the robustness of internal nodes.

In all MCMC analyses three heated chains and one single cold chain were employed, and runs were initiated with random trees. Two independent MCMC runs were conducted with six million generations per run; trees (and parameters) sampled every 100 generations. Stationarity was assessed by examining the average standard deviation of split frequencies and the Potential Scale Reduction Factor [32]. For each run, the first 25% of sampled trees were discarded as burn-in.

Following Huchon *et al.* [15] conclusions about *Laonastes* phylogeny, our Cytb and GHR phylogenetic trees were rooted with two members of the Ctenodactylidae family (*Massoutiera mzabi* and *Ctenodactylus vali*) and two Hystricognathi (*Heterocephalus glaber* and *Thryonomys swinderianus*). As no BFIBR sequences were available for these species, we rooted our BFIBR tree with another member of the Ctenodactylidae family (*Ctenodactylus gundi*) and one Hystricognathi (*Heliophobius argenteocinereus*).

### Population genetics analyses

Haplotype diversity for each gene was represented using a haplotype network. Prior to this analysis, the existence of heterozygous positions for the two nuclear genes fragments was investigated and an input file constructed from this information using SeqPHASE [33]. The phase of each haplotype and its reconstruction were carried out using PHASE v.2.1.1 [34], [35] by running the formerly built input file and by considering the default parameters of the software. This data file was used for the construction of a median-joining haplotype network, using the software NETWORK v4.500 [36].

We applied the SAMOVA procedure (Spatial Analysis of MOlecular VAriance, [37] to identify groups of geographically adjacent populations that were maximally differentiated based on sequence data. Using the computer program SAMOVA 1.0, we performed analyses based on 100 simulated annealing steps and examined maximum indicators of differentiation (*F*_CT_ values) when the program was instructed to identify K = 2 through K = 14 partitions of populations. We analysed population structure with analysis of molecular variance (AMOVA) in Arlequin 3.5.1 [38]. AMOVA divides the total variance into additive components, that is, variation within populations, among populations within groups and among groups. A population was defined as all individuals coming from one geographical locality; and groups of populations were defined according to the results of the SAMOVA analysis.

Average percentages of pairwise differences (Kimura 2-parameter) between Cyt *b* clades were calculated in Arlequin 3.5.1 [38].

In order to determine if each obtained mtDNA clade can be treated as a different population, we applied the *Snn* test [39] on each nuclear gene fragment to test for genetic differentiation between the obtained mt DNA clades. Calculation of the P-value of the *Snn* was performed with a permutation test with 1000 replicates implemented in the software DnaSP v.5 [40].

Where applicable, we estimated several statistics to describe and compare the major clades supported by our phylogenetic analyses. Number of haplotypes, number of polymorphic sites, nucleotide diversity, haplotype diversity and average number of nucleotide differences were calculated for all clades with more than 10 individuals using DNASP v5 [40]. We also applied the Tajima's D [41], Fu and Li F* and Fu and Li D* [42] neutrality tests to each nuclear and mtDNA lineage using the same software. The significance of these tests was calculated using 10,000 coalescent simulations.

The plausibility of an isolation-by-distance scenario was explicitly tested by performing Mantel's tests [43] using Arlequin 3.5.1 [38] to analyze the relationships between genetic (mean number of pairwise nucleotide differences) and geographical distances between sampling localities. The geographical distances between localities were calculated from GPS coordinates in the software GENALEX v6 [44]. Data were permuted 1,000 times to estimate the 95% upper tail probability of the matrix correlation coefficients.

### Time of divergence

Using the clades identified in the phylogenetic analyses, a Bayesian MCMC approach was used to calculate the time since genes were last shared (the most recent common ancestor, TMRCA). TMRCA can be used as a proxy for ancestral population age. Clades were dated using BEAST v1.4.6 [45]. As a calibration point we used the split between the Ctenodactylidae and *Laonastes*, estimated at 44.3±3.5 Mya [15]. A Bayesian skyline coalescent model of population size was specified [46]. Divergence times and their credibility intervals were estimated using a relaxed clock model with branch rates drawn from an uncorrelated lognormal distribution. Two independent runs of 10 million generations each, with burning of 1 million generations, were performed and later, they were combined in Tracer version 1.4 [47], which provides options for examination of the effective sample size values and frequency plots, in order to check if the mixing of the MCMC chain was adequate. Samples from both runs were combined using the software LogCombiner v.1.4.7 (available in the beast package), and a consensus chronogram with node height distribution was generated and visualized using TreeAnnotator v.1.4.7 (available in the BEAST package) and FigTree v.1.3.1 [48].

## Results

### mtDNA

**Figure 2:**
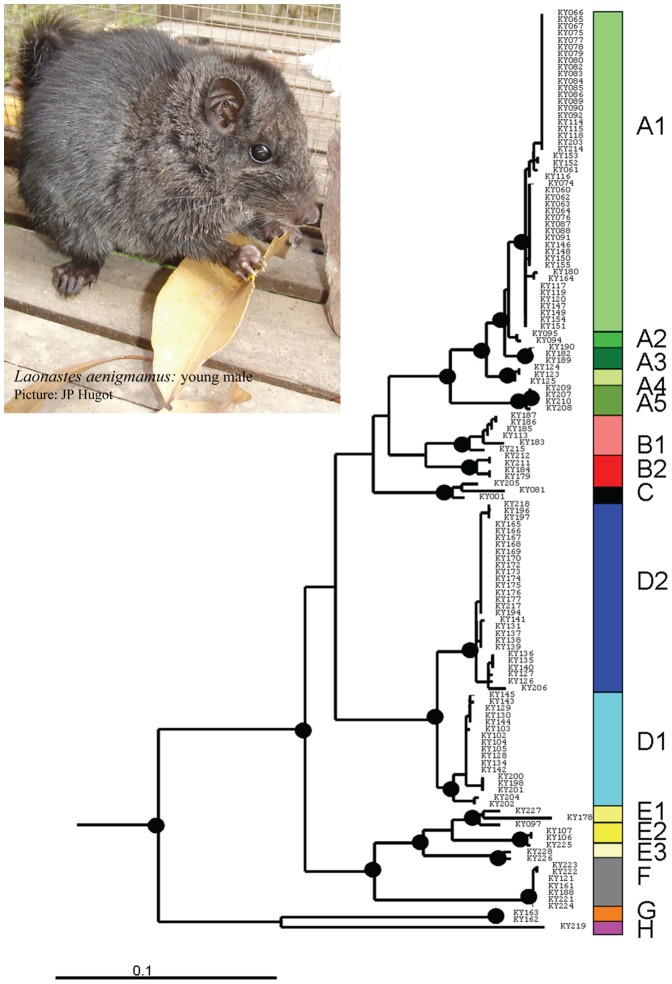
Phylogenetic relationships between Cytb sequences recovered by maximum likelihood (ML) using the GTR+I+G substitution model. Main clades are identified on the right border. Black dots represent highly supported nodes (ML Bootsrap>90, MP Bootsrap>75, Bayesian PP>0.95).

**Figure 3:**
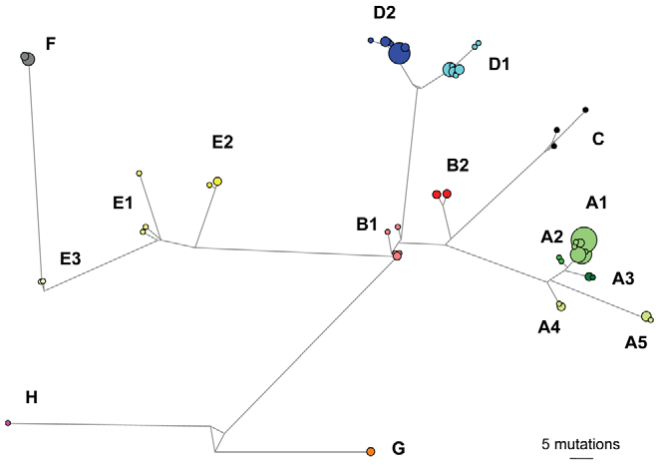
Minimum spanning network of *L. aenigmamus* mtDNA (Cyt *b*) haplotypes. Circle sizes are proportional to the number of similar haplotypes observed in the data set. Branch lengths are proportional to the number of mutations between haplotypes. Capitalized letters refer to the clade and subclades names.

**Figure 4:**
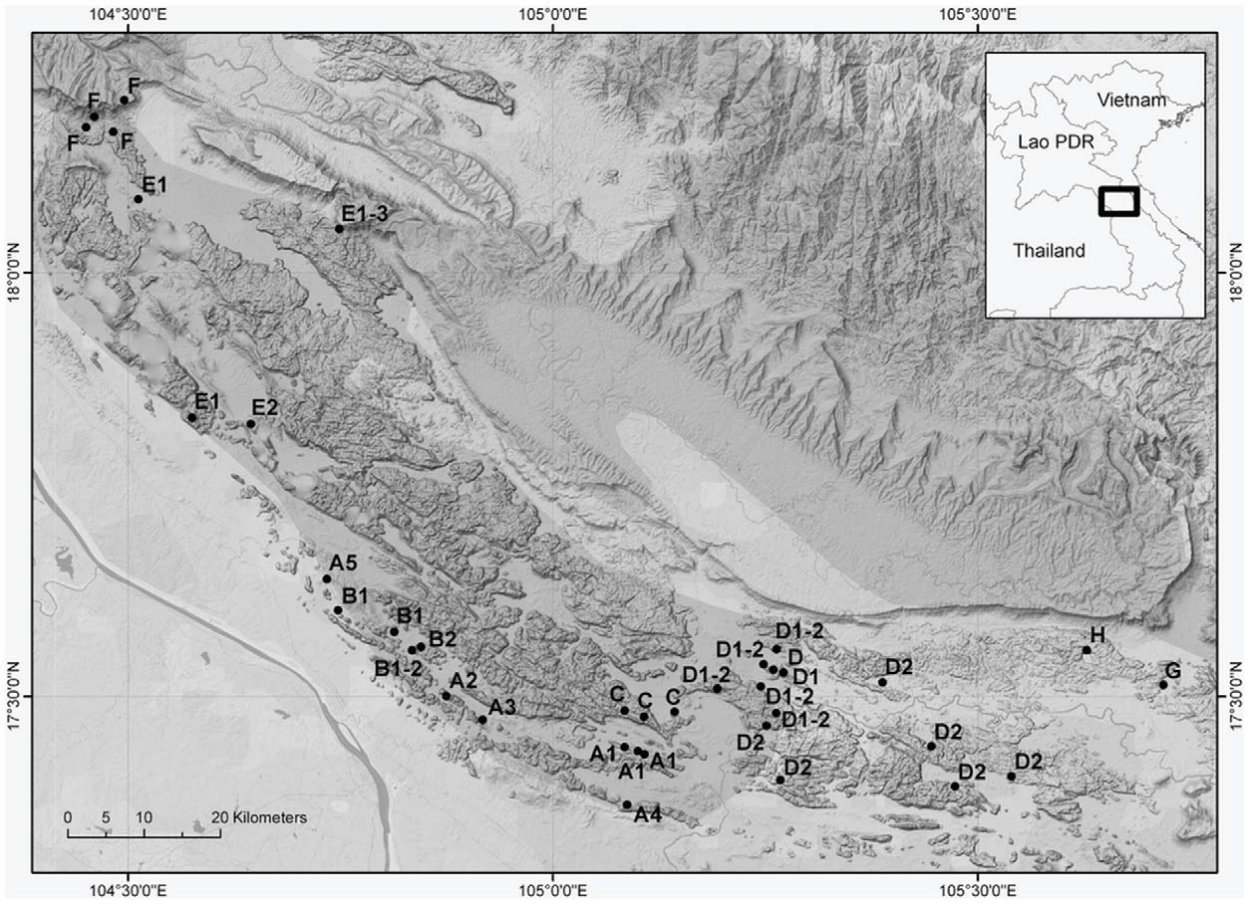
Map of sampling points showing the distribution of principal phylogenetic clades identified on the basis of mtDNA analyses.

A fragment of 983 bp of the Cyt b gene was retained for our final analyses. Among the 135 sequences analyzed, 55 different haplotypes with a total of 286 polymorphic sites can be identified. The average number of nucleotide differences is 50.56. The haplotype and nucleotide diversities are high: 0.951±0.009 and 0.051±0.002, respectively. The results of the MP, ML, Bayesian and network analyses are congruent (Fig. 2 and 3) and highlight the existence of several well-supported clades within *L. aenigmamus*. Our phylogenetic analyses reveal a strong phylogeographical structure: specimens from a single clade always originate from adjacent localities on the map (Fig. 4), and clades have allopatric geographical distributions. Eight main clades (A to H), some of them being further divided into several subclades, are identified mainly corresponding with distinct karst blocks:

Subclades A1 to A5 form a highly supported clade. Subclades A1, A2, A3, A4 and A5 are restricted to the localities of Phon Savanh-km1-3-5, Thamel, Ban Thok, Ban Nasae and Ban Nam Dik (site 2) respectively. Subclades A1, A2 and A3 form a highly supported clade, and this clade is the sister clade of A4. A5 is the sister clade of all other A subclades.
Subclades B1 and B2 are sister clades in all our analyses, but this clade is not well supported. Subclade B1 groups all specimens coming from Ban Nam Dik (site 1), and Ban Phalem and some specimens from Ban Muang. Subclade B2 is restricted to the locality of Ban Muang.
Clade C groups all specimens coming from Ban NaTung, Kohk Sawang (site 1) and Phon Savanh North.
Subclades D1 and D2 are sister clades and have sympatric distributions. Subclade D1 groups specimens coming from Ban Thapa (sites 1 and 2), Ban Phon Saet (sites 1 and 2), Khok Sawang (site 2), Ban Dang (site 1). Subclade D2 groups specimens coming from Ban Thapa (sites 1 and 2), Ban Phon Saet1, Khok Sawang (site 2), Ban Dang (sites 1 and 2), Ban Mann (sites 1, 2 and 3), Ban Natangchaye and a collecting site between Ban Thong Kong and Ban Phonkeo.
Subclades E1 and E2 are sister clades, and this clade is the sister clade of subclade E3. Subclade E1 groups all specimens from Ban Phakonko and some specimens from Ban Muangueua. Subclade E2 and E3 are restricted to the localities of Ban Na Muang and Ban Muangueua respectively.
Clade F groups all specimens coming from the northernmost localities: Ban Koun Gneun (sites 1 to 3) and Nahin.
Clade G is restricted to the locality of Ban Vong Khon.
Clade H is restricted to the locality of Ban Thalak.

Phylogenetic resolution between clades is low. Only two well-supported nodes are recovered, the first one grouping A1-A2-A3-A4-A5-B1-B2-C-D1-D2-E1-E2-E3-F and the second one grouping E1-E2-E3-F.

Average percentages of pairwise differences (Kimura 2-parameter) between clades varied from 5% between clades B and C to almost 14% between clades A and H (Table 2). Average percentages of pairwise differences between subclades vary from 1% to 4.5% (Table 3). Within clades, average percentages of pairwise differences vary from 0 to 3%, and within subclades it varies from 0.1 to 1.8%.

**Table 2:** Average percentage of pairwise differences (Kimura 2-parameter) within and between clades (A–H) identified in the phylogenetic analysis, and within and between subclades (A1–A5, B1–B2, D1–D2, E1–E3).

	A	B	C	D	E	F	G	H
A	1.02	5.32	5.81	7.07	8.89	8.98	12.01	13.74
B		1.88	4.92	5.63	7.32	8.26	11.58	12.83
C			1.31	6.05	7.74	8.62	12.49	12.98
D				1.13	7.83	8.44	12.38	13.23
E					3.14	6.72	13.07	13.17
F						0.05	13.00	13.05
G							0.00	9.95
H								0.00

**Table 3:** Average percentage of pairwise differences (Kimura 2-parameter) within and between subclades A1–A5, B1–B2, D1–D2, E1–E3.

	A1	A2	A3	A4	A5	B1	B2	D1	D2	E1	E2	E3
A1	0.29	0.91	1.41	1.97	3.89							
A2		0.10	1.01	1.57	3.53							
A3			0.07	1.79	3.83							
A4				0.07	3.38							
A5					0.10							
B1						0.58	3.06					
B2							0.41					
D1								0.40	1.97			
D2									0.33			
E1										1.79	3.40	4.02
E2											0.14	4.46
E3												0.61

Nucleotide and haplotype diversities of the main clades are given in Table 4. Neutrality tests were never significant, except the Tajima's D test for the D2 clade (Table 4).

**Table 4:** Diversity and neutrality estimates for the main clades and subclades identified in the phylogenetic analyses.

	N	Np	Nh	Hd	Pi	k	Tajima's D	Fu & Li D*	Fu & Li F*
Cytb									
A	59	61	17	0.823±0.037	1.01±0.193	9.829	−0.858	1.316	0.570
A1	47	9	8	0.724±0.047	0.286±0.000	1.896	0.385	−0.258	−0.055
B	10	31	6	0.933±0.062	1.829±0.241	13.311	1.168	0.714	0.934
D	45	48	17	0.877±0.036	1.109±0.078	10.901	−0.095	−0.823	−0.641
D1	17	17	8	0.842±0.066	0.398±0.104	3.912	−0.866	0.386	0.037
D2	28	21	9	0.733±0.081	0.33±0.063	3.249	**−1.413**	−2.161	−2.260
GHR									
A	46	9	11	0.780±0.053	0.305±0.031	2.614	0.432	0.196	0.321
A1	26	9	8	0.803±0.056	0.325±0.043	2.778	0.197	0.374	0.375
B	18	15	11	0.928±0.04	0.470±0.051	4.026	−0.291	−1.108	−1.013
D	54	6	5	0.614±0.047	0.115±0.018	0.987	−0.627	−0.574	−0.693
D1	24	5	4	0.634±0.058	0.116±0.027	0.993	−0.749	−0.503	−0.665
D2	30	4	4	0.563±0.088	0.109±0.026	0.936	−0.188	1.058	0.806
BFIBR									
A	48	10	10	0.702±0.053	0.324±0.036	2.247	−0.008	−1.033	−0.822
A1	26	10	8	0.698±0.091	0.309±0.070	2.148	−0.591	−1.174	−1.165
B	18	4	4	0.608±0.086	0.130±0.035	0.902	−0.673	−0.701	−0.797
D	54	12	13	0.693±0.065	0.458±0.061	3.177	0.603	0.376	0.535
D1	24	10	8	0.826±0.051	0.616±0.048	4.272	**1.986**	**1.408**	**1.838**
D2	30	9	8	0.510±0.109	0.197±0.055	1.366	−1.240	−1.542	−1.694

Number of specimens (*N*), number of polymorphic sites (*Np*), number of haplotypes (*Nh*), haplotype diversity (*Hd*), nucleotide diversity (*Pi*, expressed as percentages, i.e. 0.001 = 0.1%), average number of nucleotide differences (*k*). Significant values are in bold.

Results from the SAMOVA analyses showed, as expected [37], that when the number of groups of populations (K) increased, the value of *F*_CT_ increased. *F*_CT_ only increases in small increments for K = 6 to K = 14, suggesting that adding extra groups only moderately improved the model of population structure. The group membership at K = 6 is highly congruent with the groups found in the phylogenetic and network analyses: these 6 groups correspond to clades A, B+C, D, E, F, G+H. Genetic structure was highly structured among these groups of populations (AMOVA; permutation tests highly significant, *P*<0.00001), meaning that most of the genetic variability was partitioned among groups (77.28%), and only a small proportion of the variability was partitioned among populations within groups (14.71%) or within populations (8.003%). The configuration defined for K = 6 is preserved for K = 7 to K = 14, meaning that barriers are incrementally added one after the other. Clades B and C are separated for K = 8, and clades G and H are separated for K = 9.

**Figure 5:**
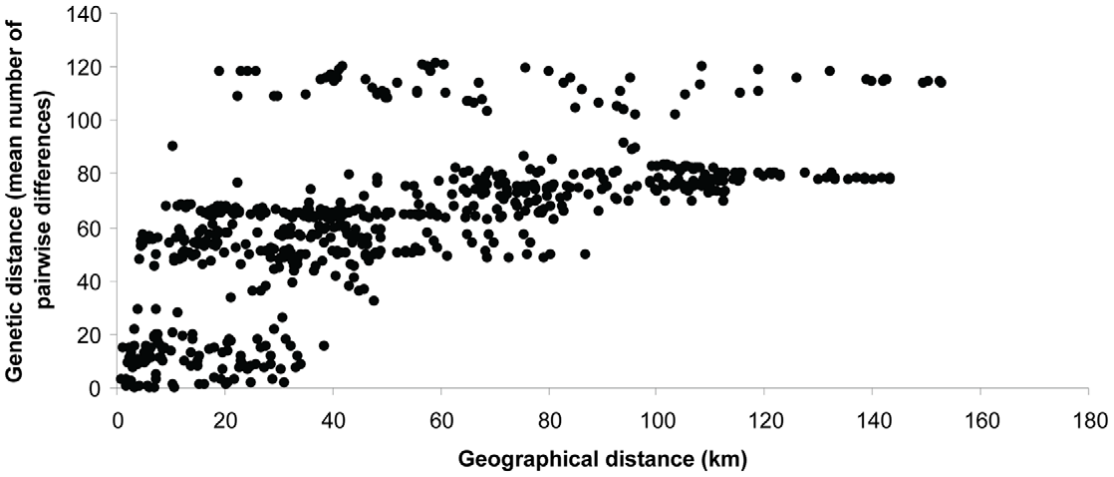
Scatter plots of geographical distances and genetic distances.

A significant correlation between geographical and genetic distance is observed (Mantel tests, P<0.0001, r = 0.625; Fig. 5) and, as expected, the genetic distance between geographically distant populations is always high. Conversely, high genetic differentiation variability may also be observed between neighboring populations. Figure 5 shows that for a small geographical distance (less than 39 km), the genetic distance can be either low (0 to 40; corresponding to intra-clade differences) or high (up to 118 pairwise nucleotide differences). Specimens coming from localities distant by less than 5 km from one another may differ by up to 58 nucleotide differences: between Phon Savanh North and Phon Savanh-km1. Whatever the geographical distance (19 to 153 km), specimens coming from the localities of Ban Vong Khon (clade G) and Ban Thalak (clade H) always differ by, at least, 90 nucleotide from all other specimens.

**Figure 6:**
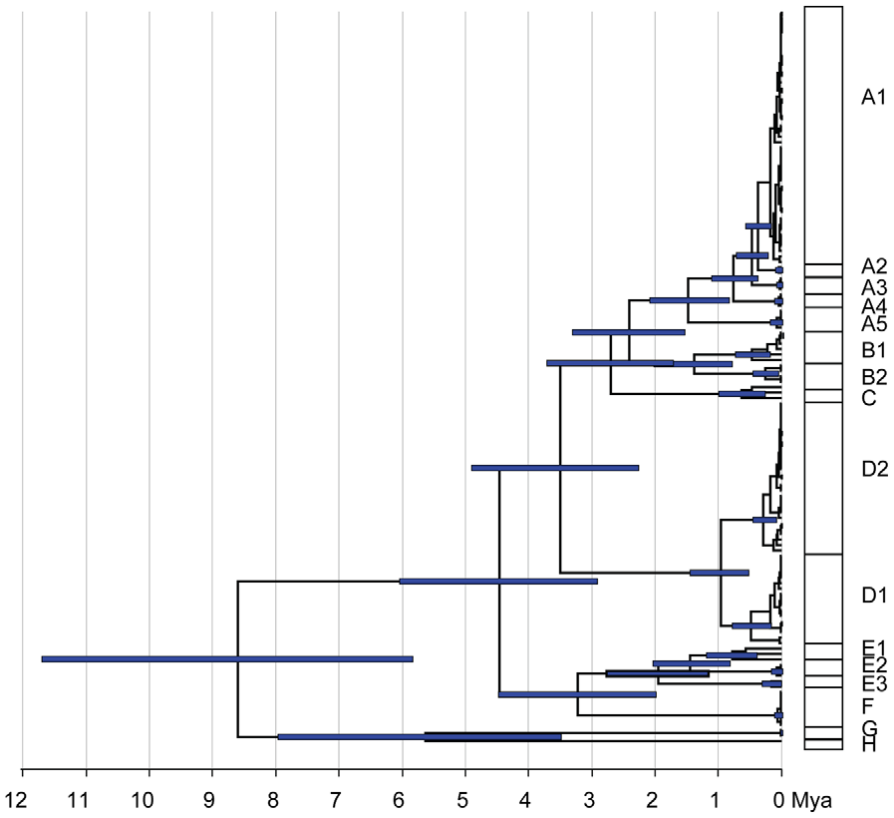
Maximum clade credibility chronogram of the Cyt *b* dataset. It was derived from an analysis in BEAST using the GTR+I+G model and a relaxed clock. Node bars indicate 95% HPD age ranges.

The TMRCA of all *Laonastes* sequences is estimated at 8.58 (range 5.84–11.61) Mya (Fig. 6). The TMRCA of clade E is 1.94 (1.20–2.79) Mya, and those of E–F is 3.20 (2.00–4.50) Mya. The TMRCA of most clades is recent (less than 1.5 Mya).

### nDNA

A total of 83 specimens, including representatives from all the defined mtDNA lineages, were sequenced for the BFIBR gene. A total of 80 specimens, including representatives from all but one (no specimen of clade H could be amplified) of all the mtDNA clades were sequenced for the GHR gene. The obtained fragments have a length of 694 bp (34 polymorphic sites for the ingroup sequences) for BFIBR and 856 bp (41 polymorphic sites for the ingroup sequences) for GHR. Heterozygous specimens are found for both gene fragments. Overall nucleotide variation in the nuclear markers is low. This low level of sequence divergence results in unresolved phylogenetic trees (Figure S1): Only clade H is supported for BFIBR sequences and clade G is supported for GHR sequences.

**Figure 7:**
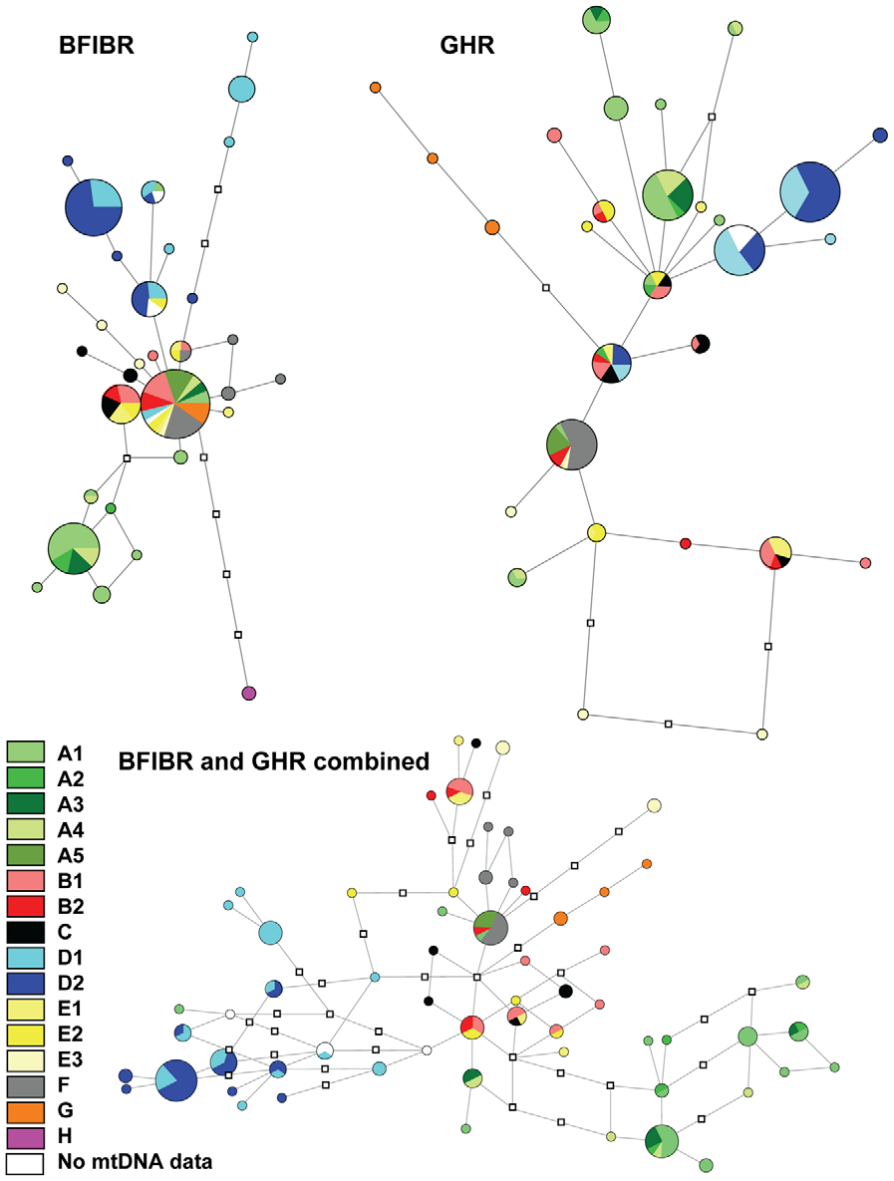
Minimum spanning networks of *L. aenigmamus* nDNA haplotypes (BFIBR, GHR and BFIBR+GHR combined). Circle sizes are proportional to the number of similar haplotypes observed in the data set. Branch lengths are proportional to the number of mutations between haplotypes. Colors refer to each clade or subclade identified in the phylogenetic analysis. Missing haplotypes are represented by a white square.

In the median joining networks clade H is well separated for BFIBR gene, and clade G is well separated for the GHR gene (Fig. 7). Moreover clades A1, A2, A, B1, C, D, D1, D2, E1, E3 and F all have one or several privative haplotypes for BFIBR, and the same is true for clades A1, A, B2, B1, D1, D2, D, E1, E2 and E3 for the GHR. When the two nuclear genes are combined (Fig. 7) resolution is better (only specimens for whom both genes were sequenced are included in the network, thus clade H is not represented): all mtDNA clades harbor one or several privative haplotypes (except clade A5), and clades A1–A2–A3–A4, D1–D2 and G are well separated.

The results of the Snn test (Table 5) indicate that the clades defined by mtDNA are significantly differentiated for both nuclear genes (except clades B and E). Subclade differentiation could only be tested between D1 and D2 and is significant for BFIBR only. All clades were therefore treated as different populations and demographic analyses were performed independently for those with appropriate sample size.

**Table 5:** Nuclear genetic differentiation of GHR and BFIBR between obtained mtDNA clades and subclades using the Snn test.

Clades	GHR		BFIBR	
A–B	0.87	***	0.79	***
A–D	0.98	***	0.94	***
A–E	0.86	***	0.86	***
A–F	0.88	***	0.84	**
B–D	0.93	***	0.93	**
B–E	0.46	ns	0.54	ns
B–F	0.89	***	0.61	*
D–E	0.95	***	0.90	**
D–F	1.00	***	0.93	***
D1–D2	0.53	ns	0.61	**

The P-value of Snn is the probability obtained by the permutation test with 1000 resplicates. ns = not significant,

* = 0.01<P<0.05,

** = 0.001<P<0.01,

*** = P<0.001.

Nucleotide and haplotype diversities are given in Table 4. Neutrality test are never significant, except for the D1 clade and BFIBR.

## Discussion

### Comparison of mtDNA and nDNA results

The high genetic divergence observed between mitochondrial lineages, is not fully corroborated by nDNA genes. However, given that: (1) private haplotypes are found for all clades, (2) Snn tests indicate that mtDNA defined clades are also significantly differentiated for both nuclear genes, (3) pattern of differentiation between clades is clearer when both nDNA genes are combined, and (4) between clade divergence events mostly occurred recently (during the Pleistocene); incomplete lineage sorting of the nuclear markers could explain the low genetic differentiation observed in nDNA. The higher evolutionary rate of mtDNA compared to nDNA, and its effective population size being one-quarter that of nuclear markers, allow a chance of recovering the pattern and tempo of recent historical events [49].

An alternative possibility would be that observed nuclear marker patterns could be due to a male-biased gene flow, with recurrent dispersion of male and rare dispersion of females leading to a contrasting pattern of nucleotide diversity between mtDNA and nDNA. Nothing is known on the mating and dispersion behavior in this species. However observations in the field, as well as metabolic and anatomical peculiarities of the species suggest that it is restricted to the karst habitat, with a low probability of survival if attempting to cross extensive open areas [50]. Thus male dispersion from one karst tower to another is doubtful, and it seems unlikely that a sex-biased gene flow could alone explain the contrasting pattern of differentiation observed between mtDNA and nDNA.

A third explanation would be that a selective sweep of the mtDNA occurred, as already been shown in various animal groups [51]–[55]. If a mitochondrial selective sweep occurred we should obtain significant results for the neutrality tests only for the mtDNA gene, and a lower nucleotide diversity of the mitochondria compared to the nDNA. This was never observed in our dataset (Table 4), suggesting that a selective sweep cannot explain the contrasting pattern of sequence divergence observed between mtDNA and nDNA.

### Taxonomy

The phylogenetic analysis of Cyt *b* revealed at least 8 well-defined clades within *L. aenigmamus*, differing by 5% to 14% K2P sequence divergence. These values are similar to those observed between most mammalian congeneric species [56], [57]. According to Baker and Bradley [57] populations that are distinguished by such genetic distance values merit further study using other molecular loci or methods to determine if an unrecognized species diversity exist. In fact they may represent distinct species under the genetic species concept (a genetic species is defined as a group of genetically compatible interbreeding natural populations that is genetically isolated from other such groups). Our nDNA data also indicate some level of genetic difference between clades. Thus, *Laonastes* could represent a complex of species. This hypothesis should now be subject to corroboration assessment before we can describe and name them.

*Laonastes aenigmamus* was described from the vicinity of Ban Muang and Ban Doy, Thakhek District (17.562°N, 104.819°E). Twelve paratypes were also collected near the village of Ban Muang, and two were collected from Thakhek market. Incomplete Cyt *b* sequences are available in Genbank for three of them: one from Ban Muang (specimen BMNH 1998.25, Genbank AM407933) and two from Thakhek market (BMNH 1998.409 and BMNH 1998.410, Genbank DQ139932 and DQ139933 respectively). When these sequences are included in our phylogenetic analyses, specimen from Ban Muang clusters unambiguously with clade B2, while specimens from Thakhek market unambiguously cluster with clade C. Thus, according to the collection locality of the holotype, the name *L. aenigmamus* should be retained for specimens of clade B2. Additional morphometric and nuclear data (microsatellites) are needed to definitively conclude on the species-specific status of all other clades. Specimens used in this study are housed at the Museum National d'Histoire Naturelle (Paris, France) and available for additional morphometric and/or molecular analyses (see Table S1 for more details). Multivariate morphometrical analyses of the skulls of all specimens, including the specimens sequenced in this study and the type specimens housed at the BMNH, are needed to investigate intra- and inter-population morphometrical variability. This will enable us to test morphometrical differentiation between clades and to potentially assign all type specimens to the corresponding clade. Additional molecular data (nuclear DNA sequences, microsatellites) will enable us to further investigate historical and ongoing gene flow between populations. All these data should allow us to evaluate the taxonomic significance of the genetic differentiation observed between clades and to frame it in formal zoological nomenclature.

### Geographical distribution

The Khammuan Limestone forms a belt of karsts 290 km long and 30–120 km wide that stretches NW-SE across the full width of Central Laos [58], [59]. This formation is isolated from other limestone, either in northern or southern Laos, by wide plains. To the West, the area is lined by the Mekong River and, to the East, by the Truong Son Mountains, which form the border between Laos and Vietnam. Since 2006, we performed numerous field studies in different provinces in Lao PDR and in Northern Thailand. During this period we questioned villagers about the presence or absence of *L. aenigmamus*. The Khammuan Limestone area was surveyed with particular attention (Fig. 1). Any time we were moving away from this area, the villager's responses to our questionnaire were becoming progressively negative. When asking people about the presence of *Laonastes* in small limestone formations peripheral to the central karst area, the answer was that *Laonastes* was present in the past but is now extinct. Thus, our surveys suggest that *L. aenigmamus* is only present in Khammuan Limestone area, as described on Fig. 1.

### Inferred history of the karsts of the Khammuan Province

Our phylogenetic analyses show that *Laonastes* populations split into 8 main genetic lineages separated by high genetic distances, and with non-overlapping geographic distributions. Moreover within clades several subclades could be identified with either allopatric (A1 to A5, E1 and E2) or sympatric (D1 and D2) distributions. Moreover E3 and B2 both have a very restricted distribution (captured in only one locality) and were collected in sympatry with E1 and B1, respectively. Figure 4 shows that clades distribution matches karst distribution: an inland insular model for *Laonastes* population structure is observed, as previously proposed by Rivière-Dobigny *et al.* [16]. A phenomenon of isolation by distance was detected, but genetic distance may be either low or high between spatially close populations. Thus, isolation by distance, only, cannot explain all the between clade differences. Observations in the field showed that once released in an open space, this species is unable to gallop, due to its remarkable anatomical peculiarities (lumbar vertebrae and pelvis fused together; weak muscles and skeleton) [50]. Because of a low likelihood of survival, if attempting to cross extensive open area, a flat plain of a few hundred meters between two limestone blocks could be sufficient to prevent migration of the animals.

If *Laonastes* is strictly endemic to karsts of the Khammuan area, its phylogeographical history may be closely tied to the history of its habitat. According to geological studies, Khammuan limestone appeared during the Middle Triassic, and was later buried under Mesozoic sandstone. A new phase of karstification occurred at the beginning of the Tertiary [58]–[61]. Aquatic erosion and abrasion by river-borne allogenic clasts contributed to the formation of a particular landscape where limestone pinnacles and towerkarst (up to 300 m high) are prominent and delineate the margins of karstic massifs arising from alluvial plains [58]. Our data strongly suggest that *Laonastes* current distribution may result from the progressive fragmentation of a formerly panmictic population: *Laonastes* ancestor being widely distributed within the Khammuan limestone around 8–9 Mya (i.e. at the end of the Miocene). No date for karst fragmentation is given in the bibliography but, if we hypothesize that long-term isolation, lineage sorting and subsequent divergence between *Laonastes* phylogroups reflect the timing of erosion, as resulting from paleoclimatic events, a possible scenario is as following: - isolation of two south-eastern blocks (corresponding to clade G and H) during the early Pliocene; - separation of a northern block (corresponding to clades E and F) at the end of the Pliocene; - rapid fragmentation in the area corresponding to clades A, B, C and D (which relationships remain largely unresolved), mainly during the Pleistocene.

If the patterns of distribution of this species reflect the general history of the landscape, similar patterns should be discovered in other species with similar ecological constraints and a similar geographical range. The hypothesis of successive karst landscape fragmentations in Khammuan (at the end of the Tertiary or beginning of the Quaternary) may be tested through the biogeographical analysis of other endemics. Of particular interest for this type of study would be the gymnure, *Hylomys megalotis* and the murid rodent, *Saxatilomys paulinae*; both known to be endemic to the Khammuan Limestone Province [11], [12].

### Conservation

Limestone is an important raw material for construction industry and karsts face increased risks of destruction from quarrying activities. As a consequence, extinctions of limestone restricted species resulting of economic development have been recorded [8]. According to Dawson *et al.* [14] from a paleontological and phylogenetic perspective, efforts to conserve *Laonastes*, the sole survivor of a morphologically distinctive family of rodents with deep evolutionary roots in Asia, should be given the highest priority. The survival of *Laonastes* is closely linked to the preservation of its habitat. Moreover our results suggesting that distinct karst towers harbor distinct evolutionary significant unit (that may represent distinct species), it is important to protect not only largest limestone areas but also isolated limestone hills.

*Laonastes* is not the only vertebrate species known to be endemic to the Khammuan Limestone region where, very probably, many invertebrate and plant species remain to be described and the conservation of *Laonastes* habitat would also allow the conservation of numerous other species.

## Conclusion

Our analyzes of *L. aenigmamus* molecular data reveal the existence of important inter-populational differences within a restricted area (about 200 km×50 km). Limestone karsts are well-known as “arks” of biodiversity and often contain high levels of endemism. However, depending on the dispersal capacities of the taxa, the level of endemicity varies: micro-endemism (i.e. site endemism) is much more frequent in invertebrates than in vertebrates, and in cave-adapted fauna than in surface fauna [4]. Exceptional micro-endemism cases were reported for snails: among just eight selected land snail genera, a large number of species (78) were found to be site endemics (species restricted to single isolated karsts) in Peninsular Malaysia. Concerning mammals a recent study on *Leopoldamys neilli* showed strong phylogeographic structure with six allopatric genetic lineages corresponding to particular regions of Thailand. However the degree of Cytb (K2P distance) genetic divergence between these clades was much lower than those observed for *Laonastes* (1.5–7.3%) despite higher geographical distances (20–630 km) and strongly isolated kart blocs [62]. Thus, *Laonastes* distribution represents an exceptional example of micro-endemism for mammals. Furthermore, the high levels of molecular divergence observed between some populations, suggest that *L. aenigmamus* may represent a complex of species and/or sub-species.

*Laonastes* is endemic to the Khammuan Limestone National Biodiversity Conservation Area and is ecologically strongly specialized. It may represent a timeline and spatial reference model to which compare the data collected for other species endemic to this region in order to infer the geological and biological history of the limestone formation in the Khammuan Province. This increases the necessity of a strict protection of this rare animal, which cannot be achieved without the protection of its natural habitat.

## Supporting Information

Figure S1 **Phylogenetic relationships between GHR (left) and BFIBR (right) sequences recovered by maximum likelihood (ML) analysis (GTR+G and HKY+G substitution model, respectively).** Main clades are identified on the right border. Black dots represent supported nodes (ML Bootsrap>75, Bayesian PP>0.95).
(TIFF)

Table S1 **List of specimens used in this study with the field number, mt DNA genetic clades identified in the phylogenetic analyses, GenBank number and locality of origin and GPS coordinates.**
(XLS)
