# Supplementary figures and images for: A Remarkable Case of Micro-Endemism in Laonastes aenigmamus (Diatomyidae, Rodentia) Revealed by Nuclear and Mitochondrial DNA Sequence Data
Source: PLoS One. 2012 Nov 14;7(11):e48145. doi: 10.1371/journal.pone.0048145 (PMC3498270; doi:10.1371/journal.pone.0048145)

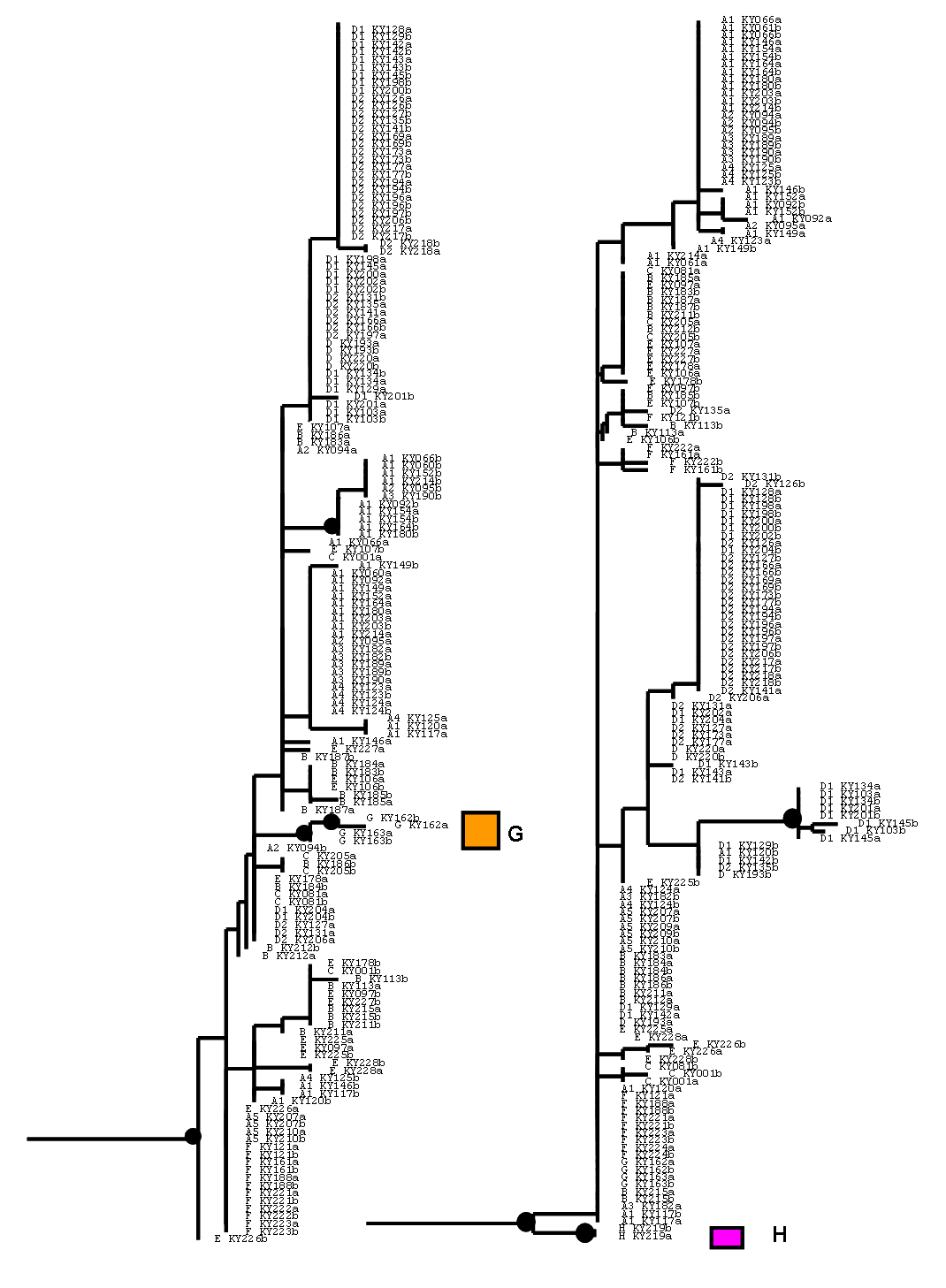

Supplement: Figure S1 — Phylogenetic relationships between GHR (left) and BFIBR (right) sequences recovered by maximum likelihood (ML) analysis (GTR+G and HKY+G substitution model, respectively). Main clades are identified on the right border. Black dots represent supported nodes (ML Bootsrap>75, Bayesian PP>0.95). (TIFF) [file pone.0048145.s001.tiff]
